# Supplementary material for: Quantitative Trait Loci for Genotype and Genotype by Environment Interaction Effects for Seed Yield Plasticity to Terminal Water-Deficit Conditions in Canola (Brassica napus L.)
Source: Plants (Basel). 2023 Feb 6;12(4):720. doi: 10.3390/plants12040720 (PMC9964187; doi:10.3390/plants12040720)
Supplement: Supplementary file 1 [file plants-12-00720-s001.zip › Supplemenatry Table S1.pdf]

Supplementary Table S1: Summary of the baseline models fitted for each trait with the variance model for genotype by environment (GE) effects, number of parameters estimated in each model in total (Total) and for the genetic variance (Genetic), log-likelihood (LogLik) and Akaike Information Criterion (AIC). DIAG: Diagonal; FA1: Factor analytic structure of order 1; FA1:FA1: Factor analytic structures of order 1 for both the between environment variance matrices of the GE additive genetic effects and non-additive genetic effects. For Baseline factor analytic linear mixed model (FALMM) and Baseline FALMM with markers specific genotype by environment (SGE) effects for water-deficit (WD) block was constrained to be zero for both additive and non-additive genetic effects. SY: Seed yield, PH: Plant height; SPAD: Chlorophyll content; DTF: Days to flower.

**Model**	**Variance model for GE effects**	**Parameters**		**LogLik**	**AIC**
		**Total**	**Genetic**		
SY					
Baseline LMM	DIAG	10	2	-4202.67	8425.34
Baseline FALMM	FA1	13	3	-4179.05	8384.10
Baseline FALMM with markers	FA1:FA1	18	6	-4165.30	8366.60
PH					
Baseline LMM	DIAG	12	2	-13695.66	27415.32
Baseline FALMM	FA1	15	3	-13640.13	27310.26
Baseline FALMM with markers	FA1:FA1	20	6	-13626.59	27293.19
SPAD					
Baseline LMM	DIAG	10	2	-2081.4	4182.94
Baseline FALMM	FA1	13	3	-2066.77	4159.53
Baseline FALMM with markers	FA1:FA1	18	6	-2051.50	4139.00
DTF					
Baseline LMM	-	6	1	-2703.91	5419.82
Baseline LMM with markers	-	7	2	-2614.90	5243.79
