# Supplementary material for: Quantitative Trait Loci for Genotype and Genotype by Environment Interaction Effects for Seed Yield Plasticity to Terminal Water-Deficit Conditions in Canola (Brassica napus L.)
Source: Plants (Basel). 2023 Feb 6;12(4):720. doi: 10.3390/plants12040720 (PMC9964187; doi:10.3390/plants12040720)
Supplement: Supplementary file 1 [file plants-12-00720-s001.zip › Supplementary Figure S1.pdf]

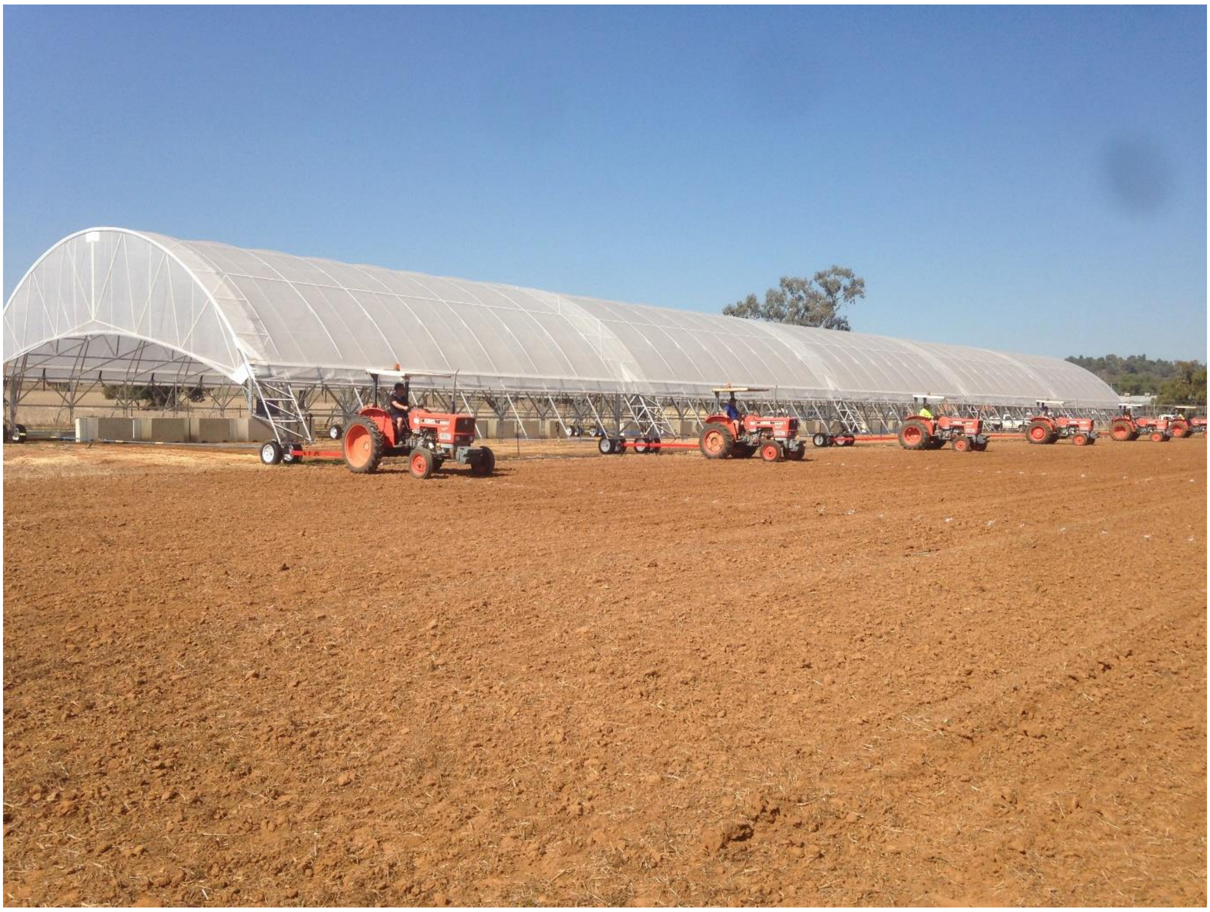

Supplementary Figure S1: Layout and movement of rainout shelter at the Wagga Wagga Agricultural Institute, NSW Department of Primary Industries, Wagga Wagga, Australia. Five rainout shelters (ROS, each was 15 m length x 15 m wide) were designed and built by Southern Irrigation Pty Ltd (Leeton, NSW, Australia). Each rainout shelter was covered with a polyethylene sheet (Polytex, Leeton, NSW, Australia) and then joined end to end. Junctions were covered with a polyethylene sheet, as shown. An array of five ROS (15 m long x 75 m wide) was hooked via a hitch with six tractors and moved to the prepared experimental site (as shown above). This 'polytunnel' was used for experimentation. Heavy-duty metal drainage ensured proper water deficit treatments. In addition, rainwater is directed away from research plots by resurfacing the area surrounding the ROS. Shelters were supported by concrete blocks (~1 ton) and the wheels locked into place to protect from strong winds. Orientation was north-south. Water-deficit block was located towards the north.
