# Supplementary material for: Quantitative Trait Loci for Genotype and Genotype by Environment Interaction Effects for Seed Yield Plasticity to Terminal Water-Deficit Conditions in Canola (Brassica napus L.)
Source: Plants (Basel). 2023 Feb 6;12(4):720. doi: 10.3390/plants12040720 (PMC9964187; doi:10.3390/plants12040720)
Supplement: Supplementary file 1 [file plants-12-00720-s001.zip › Supplementary Figure S2.pdf]

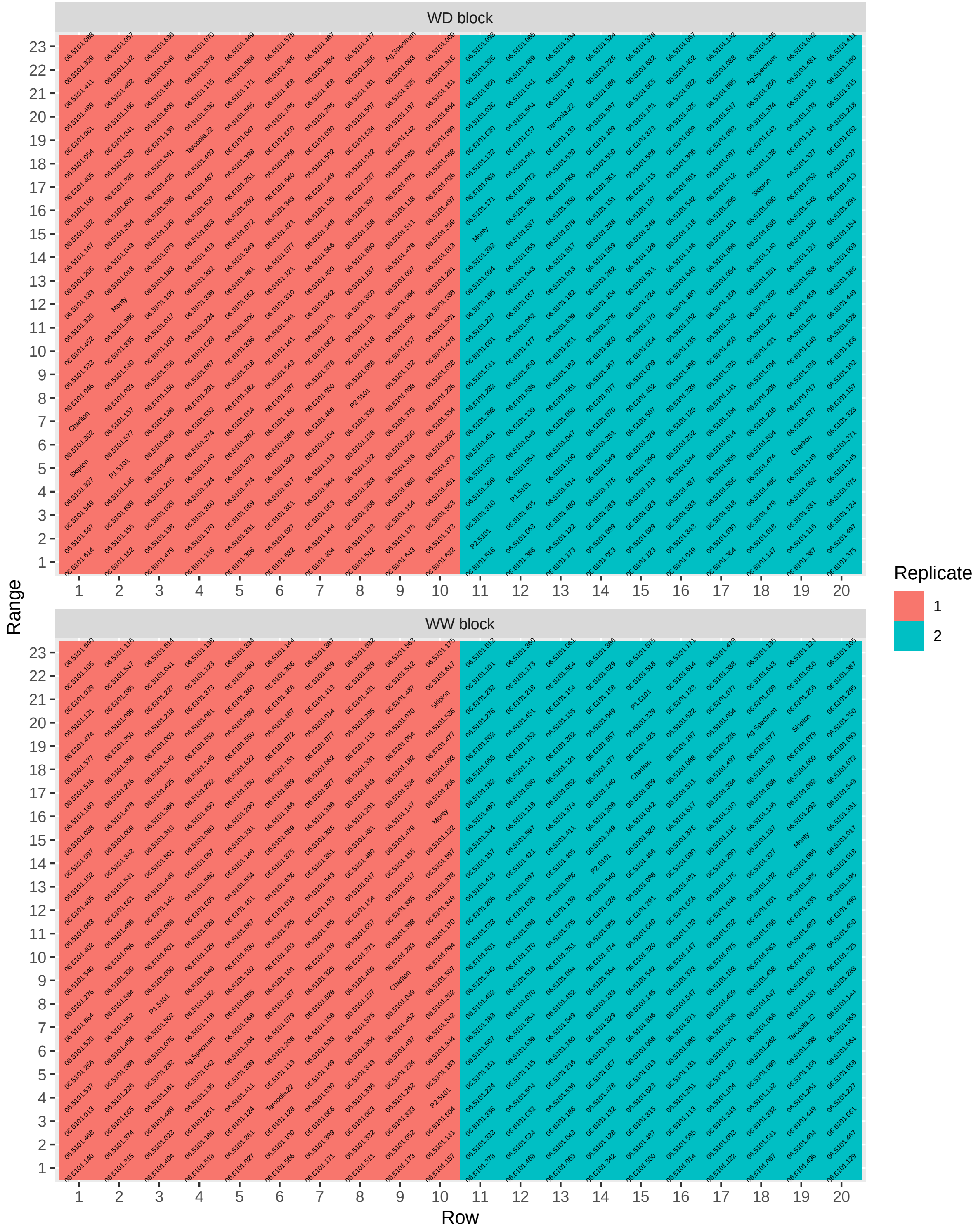

Supplementary Figure S2: Layout of the experimental design and allocation of parental lines, doubled haploid lines and check cultivars, and water regimes (WW: well-watered; WD: water-deficit).
