# Supplementary material for: Quantitative Trait Loci for Genotype and Genotype by Environment Interaction Effects for Seed Yield Plasticity to Terminal Water-Deficit Conditions in Canola (Brassica napus L.)
Source: Plants (Basel). 2023 Feb 6;12(4):720. doi: 10.3390/plants12040720 (PMC9964187; doi:10.3390/plants12040720)
Supplement: Supplementary file 1 [file plants-12-00720-s001.zip › Supplementary Method S1.pdf]

**Supplementary Method S1**

## 1. QTL analysis

The multi-environment trial (MET) dataset for each trait comprised the data on both blocks where each block is considered an 'environment'. A baseline linear mixed model (LMM) was formulated first, and then it was extended to a factor analytic (FA) form for the genotype by environment (GE) effects between environments. The GE effects were then partitioned into additive and non-additive GE effects by incorporating the marker data into the model. A working model for QTL analysis was then developed, and a genome scan was performed on this model to identify genomic regions associated with each trait. The potential markers identified from the genome scan were then thinned to establish a final multi-QTL model. Here we present information on how we developed the baseline model and the FA model fitted for each trait, depending on the current study. Raman et al., 2022 presents the details on the working model for QTL analysis, genome scan and the final multi-QTL model, as the methods and the models used are the same.

### 1.1. Formulation of the baseline LMM

Firstly, formulation of the baseline LMM commenced with fitting a model that assumed independence of the GE effects between environments. This model, termed the *diagonal* (*DIAG*) variance model for the GE effects, is analogous to analysing each environment separately. This baseline LMM was used to assess whether additional terms were required to account for non-treatment sources of variation and investigate the presence of outlier observations. Two terms were fitted as fixed effects, representing the main effects of the environments (blocks) and the interaction of the environments with those genotypes which were not genotyped but had phenotypic data (see (Tolhurst et al., 2019) for details). SY is measured for each plot, and the number of plants available in each plot was counted. The number of plants was fitted as a covariate accounting for the variation in SY (*p*-value = 0.0000000 from the Wald test) and an interaction effect with the environment (*p*-value = 0.0000000 from the Wald test). As mentioned, DTF was analysed as if both blocks constituted a single environment. Hence, the genotype main effects were partitioned into two: genotypes that were not genotyped but had phenotypic data, and genotypes that were genotyped and had phenotypic data. The former was fitted as a fixed effect, while the latter was a random one. All models also included a term representing the overall mean as a fixed effect.

The non-genetic component of the baseline LMM required the inclusion of random terms, which represent the plot (blocking) structure of the experiments as well as accounting for other significant sources of non-genetic variation which occurred in the field (random row or range terms) (see Gilmour et al. (1997) for example). Using the notation of Wilkinson & Rogers (1973), for each environment (blocks), the plot structure can be given as field plots nested within replicates (Rep/Plot). Thus the random plot structure term included in the model was Rep (Rep:Plot is the observational unit hence the errors). For PH, the baseline model also included the Rep:Plot term, which partitioned the error variance as the experimental unit of this trait is not the observational unit (see Bailey (2008)). We also allowed for variance heterogeneity between environments for all the random terms fitted in the models. For DTF, random block term was also included; for this trait, both blocks were considered a single environment. The variance models for the errors were independent and identically distributed (iid) effects with different variance parameters for each environment.

### 1.2. Factor analytic variance model for the GE effects

The baseline LMM for all traits except DTF was then extended to include FA variance models for the GE effects. A MET analysis was not necessary for DTF as this trait was measured before the water regimes were imposed for the two blocks; hence, both blocks were considered as a single environment. The FALMM estimates the genetic variances and covariances between environments using a small number of unknown factors $(k)$. These models were fitted using the reduced rank form of the FA model introduced by (Thompson et al., 2003), where common (corresponding to the common factors in the FA model) genotype by environment (CGE) effects and the specific genotype by environment (SGE) effects are modelled separately. The CGE effects may thought of as the correlated GE effects since the effects for one environment are correlated with those in at least one other environment. In contrast, the SGE effects are uncorrelated between environments. In other words, these effects are specific to the environment. The FA modelling process commences with one factor ($k = 1$) and continues until either the limit of the data is reached, or the overall percentage variance accounted for reaches 80%. For example, the limit for a MET dataset with $p = 3$ environments is $k = 1$ factor because an increase in the number of factors will result in more parameters being estimated than are possible in the fully unstructured model $(p(p + 1)/2 = 6)$. For the FA1 model, there are $pk + p - k(k - 1)/2 = 6$ parameters to estimate.

In our case, as we had only two environments (blocks), the variance of the SGE effects of the WD block was constrained to be zero following (Cullis and Smith, 2016). We refer to these models as baseline FALMM. On average, the percentage of genetic variation accounted for (%VAF) by the baseline FALMM were above 85% for all traits (SY: 92.9%, PH: 99.9% and SPAD: 85.8%). Estimated genetic correlations between environments were all greater than 0.88 for all traits.

### 1.3. Partitioning GE effects into additive and non-additive GE effects

The baseline FALMM was then extended to include marker information to determine the genomic regions influencing the traits' plasticity to terminal water stress. GE effects were partitioned into additive, and non-additive GE effects (Oakey et al., 2007) and each were modelled by separate FA variance models. We utilised a genetic linkage map of the 5101 DH population based on 1793 'bin' DArTseq markers representing all 19 chromosomes of *B. napus*. The linkage map was constructed using the package *ASMap* (Taylor and Butler, 2017) in *R* statistical computing environment (R Core Team, 2021) utilising the minimum spanning tree algorithm (Wu et al., 2008). The missing values in these markers were imputed using the *k*-nearest neighbour method (Troyanskaya et al., 2001). The resulting marker matrix, $\boldsymbol{M}$, was used to construct a genomic relationship matrix, $\boldsymbol{K}$, (GRM) (VanRaden, 2008), using the package *pedicure* (Butler, 2021) in *R* statistical computing environment (R Core Team, 2021), where the GRM was scaled by the reciprocal of the average marker variance. The GRM, $\boldsymbol{K}$, was the matrix proportional to the variance of the additive GE effects. The resulting model was equivalent to the standard genomic best linear unbiased prediction (GBLUP) model used in models for genomic selection (Tolhurst et al., 2019). We refer to this model as the baseline FALMM with markers. Supplementary Table 1 presents a summary of baseline LMM, baseline FALMM and the baseline FALMM with markers in terms of the variance model for the GE effects, the number of estimated parameters (total and genetic), residual log-likelihood and Akaike Information Criterion (AIC). The AIC value of the baseline FALMM with markers (or baseline LMM for DTF) was the lowest for each trait, indicating that this model is the better fit.

### 1.4. Investigation of GE interaction in the MET dataset

To test the significance of the specific GE interaction in the MET dataset for the three traits SY, PH and SPAD, we compared the baseline FALMM with markers, which models both the

common and specific GE interaction, with a model that only models the common GE interaction for each trait using the likelihood ratio test. The baseline FALMM with markers was reduced to model only the common GE interaction in the random component together with all the non-genetic effects fitted before. The terms in the fixed component were retained as before.

## 2. Multivariate analysis

The genetic correlations between traits were obtained using multivariate analyses. The data for all traits were combined and analysed together within the MET analysis framework using FALMM, where the traits were considered as ‘environments’. Three sets of multivariate analyses were performed: (1) all traits from WW block, (2) all traits from WD block and (3) SY from WW block and carbon isotope discrimination ($\Delta^{13}C$) from the previous study (Raman et al., 2022). As the water regimes were imposed after flowering, DTF was considered only in the first set. For brevity, we only present the details of the analyses conducted for sets 1 and 3. The analysis conducted for set 2 is very similar to set 1. We first constructed the multivariate data set and formulated the baseline multivariate LMM. It is then extended to a FA form for genotype by trait (GT) effects between traits. We then partitioned the GT effects into additive and non-additive by incorporating the marker data into the model, and finally, the inference on the genetic correlation between traits was obtained.

### 2.1. Constructing the multivariate dataset

The multivariate dataset was constructed by combining the datasets for all traits (row-wise) under evaluation. The response variable, i.e. the measurements taken for each trait, is stored in the same column. For set 1, the traits SY, PH and SPAD from WW block and DTF from one of the blocks (WW block, although there was no difference between the blocks in terms of watering) were combined. For set 3, $\Delta^{13}C$ from 2 experiments conducted in 2017 and 2018 and SY from the WW block of the current study were combined. As there were data from multiple environments for $\Delta^{13}C$, we defined a factor called ‘EnvironmentTrait’ with 3 levels which were the combinations of the levels of experiments and the 2 traits under evaluation: ‘1: $\Delta^{13}C$’, ‘2: $\Delta^{13}C$’ and ‘3: SY’. For set 1, the multivariate dataset was ordered as traits nested within observational units as all observations were measured for the same plots. In contrast, for set 3, it was ordered as observational units within each of the levels of EnvironmentTrait. We note that the multivariate datasets in our case were very similar to

MET datasets as the combined analysis of all levels of either trait or EnvironmentTrait was analogous to a MET analysis, where we consider each level of these factors as an ‘environment’.

## 2.2. Formulating the baseline multivariate LMM

For set 1, a diagonal variance model was fitted for the GT effects similar to the baseline LMM for QTL analysis. Three terms were fitted as fixed effects representing the main effects of the traits, the interaction of the trait with those genotypes which were not genotyped but had phenotypic data and the number of plants as a covariate accounting for the variation only for SY. We also included a term representing the overall mean as a fixed effect. The non-genetic component of the baseline multivariate LMM was derived from the baseline LMM of each trait used for the QTL analysis. For all random terms fitted in the model we allowed for variance heterogeneity between the traits. Considering that all traits were measured for the same field plots, error structure was specified as two-dimensional, with iid effects and an unstructured variance matrix across traits.

Similar to set 1, for set 3, we began with a diagonal variance model for the GT effects. Three terms were fitted as fixed effects representing the main effects of EnvironmentTrait, the interaction of EnvironmentTrait with those genotypes which were not genotyped but had phenotypic data and the number of plants as a covariate accounting for the variation only for SY at the corresponding environment. We also included a term representing the overall mean as a fixed effect. For SY, the non-genetic component of the baseline multivariate LMM was derived from the baseline LMM used for the QTL analysis. As $\Delta^{13}C$ was a trait measured from multi-phase experiments: field and laboratory, the random plot structure terms from both field and the laboratory phases were included in the baseline multivariate LMM. Using the notation of Wilkinson and Rogers (1973), the plot structure of the field phase of the experiment can be given as Rep/Plot. Hence, the random terms Rep and Rep:Plot were fitted in the baseline multivariate LMM only for this trait at the corresponding environments. Random row and range effects were included as necessary.

Similarly, the plot structure for the laboratory phase can be considered as sample positions (Tube) nested within carousels crossed with runs (Run*(Carousel/Tube)), given that the same carousels were used in each run. This resulted in the inclusion of the random terms Run, Carousel, Run:Carousel and Carousel:Tube (Run:Carousel:Tube is the observational

unit hence the errors). For all random terms fitted in the model, we allowed for variance heterogeneity between all levels of EnvironmentTrait. The variance models for the errors were iid effects with different variance parameters for each level of EnvironmentTrait. Moreover, we constrained the errors to be zero (very small) for the level '3:SY' of EnvironmentTrait as the errors for SY is at the field phase, and these have already been fitted as random effects. That is, we only needed to model the errors for the levels of EnvironmentTrait, which had the laboratory phase, in other words, laboratory errors.

### 2.3. Factor analytic variance model for the GT effects

The baseline multivariate LMM for both sets were then extended to include FA variance models for the GT effects. As we had four traits for set 1 and three levels of EnvironmentTrait for set 3 we fitted an FA1 model for both the sets. We refer to these models as baseline multivariate FALMM.

### 2.4. Partitioning GT effects into additive and non-additive GT effects

The baseline multivariate FALMM was then extended to include marker information. GT effects were partitioned into additive and non-additive GT effects, and each were modelled by separate FA variance models. Marker information was incorporated into the model via the GRM, $K$. We refer to this model as final multivariate FALMM with markers.

### 2.5. Inference on genetic correlation between traits

For set 1, the between trait correlation matrix for the total (additive plus non-additive) genetic effects was obtained from the final multivariate FALMM with markers. For set 3, as $\Delta^{13}C$ was measured at multiple environments (2017 experiment and 2018 experiment), we first obtained the between EnvironmentTrait variance matrix for the total genetic effects. The values in this matrix were averaged across the two environments for $\Delta^{13}C$ using a transformation matrix, and consequently, between trait genetic correlations were obtained.

## References


Bailey, R. (2008). Design of Comparative Experiments (Cambridge Series in Statistical and Probabilistic Mathematics). Cambridge: Cambridge University Press. doi: 10.1017/CBO9780511611483 Gilmour, A. R., Cullis, B. R. and Verbyla, A. P. (1997). Accounting for natural and extraneous variation in the analysis of field experiments. *Journal of Agricultural, Biological, and Environmental Statistics* 2, 269–293. doi: 10.2307/1400446

Butler, D. (2021). Pedicure: pedigree tools. R package version 2.0.1. Available online at: https://mmade.org/pedicure/.

Cullis, B.R., and Smith, A.B. (2016). The analysis of QTL and QTL by treatment experiments using spatial models for marker effects. Technical report, University of Wollongong, Australia.

Oakey, H., Verbyla, A., Cullis, B.R., Wei, X., and Pitchford, W. (2007). Joint modelling of additive and non-additive (genetic line) effects in multi-environment trials. *Theoretical and Applied Genetics* 114, 1319–1332.

R Core Team (2021). 'R: A language and environment for statistical computing.' *R Foundation for Statistical Computing: Vienna, Austria*. https://www.R-project.org/

Raman, H., Raman, R., Pirathiban, R., Mcvittie, B., Sharma, N., Liu, S., Qiu, Y., Zhu, A., Killian, A., Cullis, B., Farquhar, G.D., Williams, H.S., White, R., Tabah, D., Easton, A., and Zhang, Y. (2022). Multi-environment QTL analysis delineates a major locus associated with homoeologous exchanges for water-use efficiency and seed yield in allopolyploid *Brassica napus*. *PLant Cell and Environment*, 45, 2019– 2036. https://doi.org/10.1111/pce.14337

Taylor, J.D., and Butler, D. (2017). R Package ASMap: Efficient genetic linkage map construction and diagnosis. Journal of Statistical Software, 79(6), 1-29. doi:10.18637/jss.v079.i06.Thompson, R., Cullis, B., Smith, A., and Gilmour, A. (2003). A sparse implementation of the average information algorithm for factor analytic and reduced rank variance models. *Australian and New Zealand Journal of Statistics* 45, 445-459.

Tolhurst, D.J., Mathews, K.L., Smith, A.B., and Cullis, B.R. (2019). Genomic selection in multi-environment plant breeding trials using a factor analytic linear mixed model. *Journal of Animal Breeding and Genetics* 136, 279-300.

Troyanskaya, O., Cantor, M., Sherlock, G., Brown, P., Hastie, T., Tibshirani, R., Botstein, D., and Altman, R.B. (2001). Missing value estimation methods for DNA microarrays. *Bioinformatics* 17, 520–525. doi: 10.1093/bioinformatics/17.6.520.

VanRaden, P. M. (2008). Efficient Methods to Compute Genomic Predictions. *Journal of Dairy Science*, 91, 4414–4423. doi: 10.3168/jds.2007-0980

Wilkinson, G. N. and Rogers, C. E. (1973). Symbolic description of factorial models for analysis of variance. *Journal of the Royal Statistical Society. Series C (Applied Statistics)* 22, 392–399

Wu, Y., Bhat, P. R., Close, T. J. and Lonardi, S. (2008). Efficient and Accurate Construction of Genetic Linkage Maps from the Minimum Spanning Tree of a Graph. *PLoS Genetetics* 4(10): e1000212. https://doi.org/10.1371/journal.pgen.1000212
